# Lymphocyte homing disorder: a concealed driver of intestinal immune collapse in sepsis

**DOI:** 10.3389/fimmu.2026.1827322

**Published:** 2026-05-28

**Authors:** Huixian Geng, Dacheng Xiong, Xuechun Lü, Jing Wang, Hua Chen, Lijing Jia

**Affiliations:** 1 Hebei Medical University, Shijiazhuang, China; 2 High Dependency Unit, Hebei General Hospital, Shijiazhuang, China

**Keywords:** gut immune microenvironment, homing pathway, homing-targeted therapy, intestinal lymphocyte homing, sepsis

## Abstract

Sepsis is defined as life-threatening organ dysfunction caused by a dysregulated host response to infection. “Lymphocyte homing”—the targeted migration of lymphocytes between organs—serves as the physiological foundation for immune surveillance and local immune homeostasis. Under septic conditions, this precisely regulated process is severely disrupted, leading to failed recruitment of immune cells to infection/injury sites and exacerbated depletion of lymphoid tissues, ultimately inducing systemic immune imbalance. This review elaborates on three core aspects: the physiological basis of intestinal lymphocyte homing, the mechanisms by which sepsis disrupts this pathway (including immune microenvironment remodeling, abnormal homing signaling, and regulatory T cell dysfunction), and targeted interventional strategies (chemokine receptor antagonists, integrin-directed regulation, and microbiota-immune crosstalk modulation). Understanding lymphocyte homing disorder provides a novel breakthrough for deciphering septic immunosuppression and improving clinical outcomes.

## 1 Introduction

As the largest immune organ in the human body, the intestine relies on the dynamic balance of its mucosal immune system to maintain systemic immune homeostasis. This balance is governed by a complex “immune-gut axis” regulatory network, in which intestinal lymphocyte homing plays a key role (1). Through the precise expression and recognition of chemokine receptors such as C-C chemokine receptor 9 (CCR9) and the gut-homing integrin α4β7, this mechanism selectively guides T and B lymphocytes from the peripheral circulation to the intestinal lamina propria and Peyer’s patches (2). Peyer’s patches are specialized lymphoid tissues distributed in the mucosa and submucosa of the small intestine (especially the ileum), and constitute an essential component of mucosa-associated lymphoid tissue (MALT). Their main function is to recognize intestinal pathogens (e.g. bacteria and viruses), initiate immune responses, and maintain intestinal mucosal defense (3). Gut-specific homing not only establishes a unique intestinal immune microenvironment, but also enables fine regulation between local immune defense and systemic immune tolerance, forming a dynamic bridge connecting the intestine and the systemic immune system. During the progression of sepsis, this finely tuned homing network is severely disrupted, leading to aberrant distribution and functional dysregulation of immune cells, ultimately resulting in a vicious cycle of “persistent inflammation, immunosuppression, and catabolism syndrome” (4). In-depth analysis of the dynamic evolution of intestinal lymphocyte homing under septic conditions provides a novel breakthrough for understanding the mechanisms of immunoparalysis in sepsis (5).

## 2 Physiological basis of lymphocyte homing

Lymphocyte homing refers to the process by which lymphocytes migrate directionally from the bloodstream to specific tissues, such as the gut, skin, and lymph nodes (6). This mechanism is essential for immune surveillance, inflammatory responses, and immunological memory. Its physiological basis involves several key steps, with the core being the pairing recognition between “homing receptors” and “addressins”: vascular endothelial cells in different tissues express specific addressins that guide selective lymphocyte homing. Lymphocyte homing is a multi-step cascade (“rolling – adhesion – migration”) (7). Initially, lymphocytes transiently interact with endothelial surface glycosylated ligands (e.g., PNAd, E−selectin ligands) via selectins (e.g., L−selectin). Through these low−affinity interactions, cells “roll” along the vessel wall without immediate arrest. Subsequently, chemokines (e.g., CCL25, CCL19) secreted by the vascular endothelium bind to G protein−coupled receptors (GPCRs) (e.g., CCR9, CCR7) on lymphocytes (8, 9), activating integrins (e.g., α4β7, LFA−1). This activation induces a conformational change in integrins from a low−affinity to a high−affinity state, thereby enhancing binding to endothelial adhesion molecules (10). In this process, integrins tightly engage with addressins, and lymphocytes follow the chemokine gradient, transmigrate across the endothelium, and enter the tissue through intercellular gaps. The physiological basis of lymphocyte homing is therefore “precise receptor–ligand recognition plus chemokine gradient guidance”, which ensures efficient localization of immune cells to specific tissues. The key molecules involved in this multi−step cascade and their functions are summarized in **Table 1**.

**Table 1:** Key molecules and functions involved in the multi-step cascade of intestinal lymphocyte homing.

Step	Homing receptor	Homing ligand	Primary function
Rolling	L-selectin (CD62L)	PNAd (peripheral lymph node addressin, containing core proteins such as CD34)	Mediates initial contact and rolling, allowing lymphocytes to roll slowly along the vascular endothelium and sense local environmental signals (11–13)
	α4β7 integrin	MAdCAM-1 (mucosal addressin)	Mediates initial contact and rolling, allowing lymphocytes to roll slowly along the vascular endothelium and sense local environmental signals (14)
	CLA (cutaneous lymphocyte-associated antigen)	E-selectin	Mediates rolling homing of memory T cells to the skin (15)
	PSGL-1 (P-selectin glycoprotein ligand-1)	P-selectin	Mediates lymphocyte rolling under inflammatory conditions (16)
Activation	CCR7 (G protein-coupled receptor)	CCL21 (chemokine)	Chemokine signaling activates integrins, triggering the transition from rolling to firm adhesion (9, 17)
	CCR9 (G protein-coupled receptor)	CCL25 (chemokine)	Activates α4β7 integrin in gut homing, mediating gut-specific homing (18)
Adhesion	α4β7 integrin	MAdCAM-1 (mucosal addressin)	Mediates firm adhesion of lymphocytes to intestinal mucosal endothelium (19)
	LFA-1 (αLβ2 integrin)	ICAM-1/ICAM-2	Mediates broad firm adhesion and is a key step for lymphocyte transendothelial migration (20)
Migration	LFA-1 (αLβ2 integrin)	ICAM-1	Supports lymphocyte crawling on the endothelial surface to locate appropriate exit sites (20)
	CD44	Hyaluronate	Mediates migration of activated lymphocytes to inflammatory sites (21)

## 3 Mechanisms of lymphocyte homing disorder in sepsis

### 3.1 Sepsis remodels the gut immune microenvironment

During the pathological progression of sepsis, an uncontrolled systemic inflammatory response can trigger a “cytokine storm,” characterized by excessive release of pro−inflammatory mediators such as tumor necrosis factor-a(TNF-a) and interleukin-6(IL-6), which directly disrupt intestinal mucosal barrier homeostasis (22). This disruption manifests as:

1. significant damage to tight junction structures, with time−dependent decreases in both mRNA transcription and protein expression of key junctional proteins and the core scaffold protein zonula occludens-1 (ZO-1), leading to abnormally increased permeability between intestinal epithelial cells (23–25);
2. marked upregulation of intestinal epithelial cell apoptosis pathways (e.g., caspase-3/8 activation) (26), accompanied by suppressed expression of the anti−apoptotic protein Bcl-2, resulting in an epithelial apoptosis rate higher than under physiological conditions (27); mitochondrial reactive oxygen species simultaneously activate both apoptosis (caspase-3) and pyroptosis (gasdermin D, GSDMD), further exacerbating intestinal barrier injury (28);
3. reduced MUC2 mucin secretion and functional defects in Paneth cells, ultimately creating a vicious cycle of microbiota and endotoxin translocation along the “gut−systemic axis”-a mechanism that has been confirmed as a major driver of secondary multiple organ dysfunction in sepsis (29–32). This “leaky gut” phenomenon promotes translocation of large quantities of gut microbiota and their metabolites, triggering an excessive Toll-like receptor 4 (TLR4)-mediated innate immune response (30). Single−cell sequencing studies have shown that in early sepsis, the proportion of CD103+ dendritic cells in the intestinal lamina propria is abnormally elevated, and their secretion of CCL20 increases, creating an abnormal chemokine gradient field (33, 34).

During the progression of sepsis, the microvascular endothelial cells in the intestine undergo significant functional changes. First, endothelial activation and upregulation of adhesion molecules (E-selectin/VCAM-1) occur. These activated vascular endothelial cells act as “signal beacons”, producing large amounts of E-selectin and VCAM-1 on their surface (35). Studies have shown that higher levels of these markers correlate with more severe disease. Under normal conditions, the directional migration of immune cells (e.g., lymphocytes) is precisely guided, but these excess E-selectin and VCAM-1 molecules interfere with this guidance system, causing immune cells (particularly those bearing α4β7 integrin) to adhere incorrectly to intestinal blood vessels (36), leading to local accumulation of immune cells. Second, microcirculatory disturbances and hypoxia (HIF-1αactivation) occur. Reduced local blood flow in the intestine leads to tissue hypoxia. Under hypoxic conditions, HIF-1a protein is stabilized due to impaired degradation (37, 38), thereby activating downstream target genes and promoting a metabolic switch in immune cells (e.g., macrophages, T cells) from oxidative phosphorylation to glycolysis. This metabolic reprogramming (e.g., upregulation of glucose transporter 1 (GLUT1) and hexokinase 2 (HK2), lactate accumulation) disrupts immune homeostasis, increasing pro-inflammatory M1 macrophages and Th17 cells while decreasing anti-inflammatory M2 macrophages and regulatory T cells (Tregs). At the same time, intestinal epithelial tight junction proteins (e.g., ZO-1) are lost, and barrier function is impaired. Sustained release of inflammatory cytokines (e.g., IL-1β, TNF-α) further aggravates microcirculatory disturbances, creating a vicious cycle of “hypoxia -metabolic dysregulation -immune dysregulation” that ultimately exacerbates the progression of intestinal disease.

In septic patients, the proportion of CCR9+CD4+ T cells in peripheral blood is decreased compared with healthy controls, whereas the number of resident memory T cells in the intestinal mucosa is increased. This distribution imbalance is closely associated with dysregulated expression of gut−homing receptors, manifested as downregulation of CCR9 and α4β7 integrin expression on circulating lymphocytes, while the concentration of the CCR9 ligand CCL25 in the local intestinal milieu is elevated of physiological levels, resulting in a “homing receptor-ligand dissociation” phenomenon (36, 39).

### 3.2 Abnormal homing pathways drive immune imbalance

Sepsis is a systemic inflammatory storm triggered by infection. Its lethality stems not only from the pathogen itself but also from the self-destructive dysregulation of the immune system. By disrupting the two critical biological navigation systems—CCR9/CCL25 and a4β7/MAdCAM-1—sepsis causes fatal spatiotemporal mislocalization of immune cells and drives disease deterioration (8). Temporally, CCL25 signaling exhibits a biphasic imbalance characterized by early hyperactivity and late exhaustion. Spatially, the α4β7/MAdCAM-1 pathway manifests a liver-gut paradox, resulting in regional organ injury and even a vicious cycle between the liver and intestine, ultimately leading to systemic immune imbalance and multiple organ failure (40). Recent studies indicate that tissue-specific dysregulation of the α4β7 integrin/MAdCAM-1 pathway is the core mechanism underlying the errant navigation of immune cells (41).

#### 3.2.1 CCR9/CCL25 axis: temporal dynamic imbalance

In healthy individuals, the migration of immune cells, particularly T cells, is precisely regulated, with the CCR9/CCL25 axis serving as a key “navigation signal” for gut homing. CCL25 is primarily secreted by small intestinal epithelial cells, and the CCR9 receptor on the surface of T cells recognizes this signal, guiding their precise migration to the intestine and maintaining local immune balance (42). It is worth noting that the reported concentrations of CCL25 vary among studies, mainly due to differences in detection methods (e.g., ng/mL levels commonly used in chemotaxis assays versus pg/mL physiological concentrations), cell types, and experimental conditions. However, these variations do not affect the overall trend of dynamic changes in CCL25 during sepsis (43–46). During sepsis, this system is severely disrupted, exhibiting a “first hyperactivation, then failure” pattern: in the early phase (0–24 hours), the systemic inflammatory storm causes a surge in blood CCL25 levels, leading to an excessive influx of T cells that would normally remain in the blood or lymph nodes into the intestine (47). Such over−recruitment not only fails to effectively combat infection but also exacerbates intestinal inflammation (48). In the later phase (after 72 hours), the ability of intestinal epithelial cells to produce CCL25 is greatly diminished due to hypoxia, inflammatory injury, and other factors. Consequently, immune cells can no longer home properly to the intestine, resulting in a collapse of local immune defense, which closely mirrors the transition of septic patients from a hyper-inflammatory state to immunoparalysis (49). This mechanistic insight explains why early anti−inflammatory therapy for sepsis may be ineffective, whereas later−stage immune enhancement strategies are needed -because the homing signals differ fundamentally between phases (50). However, clinical translation targeting the CCR9/CCL25 axis still faces challenges: although CCR9 antagonists have shown good efficacy in various animal models of colitis, subsequent clinical trials (e.g., vercirnon for Crohn’s disease) failed to meet their primary endpoints, suggesting that the regulation of this axis in humans is more complex, and factors such as drug pharmacokinetics and disease heterogeneity may contribute to discrepancies between animal and clinical results. This further emphasizes the need to design time-sensitive and individualized intervention strategies that target the phase-specific changes in homing signals during sepsis (51).

#### 3.2.2 α4β7 integrin/MAdCAM-1 pathway: spatial navigation errors

In healthy individuals, the immune system achieves precise deployment of immune cells through sophisticated regulatory pathways. Among these, the integrin α4β7 is a specialized molecule on the surface of gut−homing T cells, functioning as a “biological locator” (52, 53). It specifically recognizes MAdCAM-1 on the surface of intestinal vascular endothelial cells, forming a matching pair (41). This molecular docking mechanism ensures that T cells bearing α4β7 (especially CD4+ T cells that regulate intestinal immunity) migrate directionally to the intestinal mucosa, where they perform immune surveillance, pathogen clearance, and tissue repair (54, 55). The precision of this process is reflected in both tissue specificity and dynamic regulation: MAdCAM-1 is predominantly expressed on intestinal vascular endothelial cells, with extremely low basal expression in other organs such as the liver (56). Upon intestinal infection or injury, local inflammatory signals (e.g., TNF-α, IL-1β) can transiently upregulate MAdCAM-1 expression to rapidly recruit additional immune cells for support (14, 56). However, in the context of sepsis “a systemic inflammatory state” the dynamic changes of the α4β7/MAdCAM-1 pathway remain controversial. On the one hand, it is widely accepted that this pathway is upregulated by pro-inflammatory cytokines (e.g., TNF-α) and downregulated by anti-inflammatory cytokines (e.g., IL-10), but its precise spatiotemporal changes in different phases and tissues during sepsis have not been uniformly defined (57, 58). Recent studies have revealed that the interaction between α4β7 and MAdCAM-1 is regulated by “catch bond” mechanics, and the hemodynamic alterations accompanying sepsis may profoundly affect this process, although the exact patterns of change remain unknown (59). These controversies indicate that the α4β7/MAdCAM−1 pathway exhibits complex, temporally and spatially heterogeneous, tissue−specific regulation in sepsis.

In the early phase of sepsis, systemic inflammatory factors (e.g., LPS, IL−6) trigger aberrant activation of liver vascular endothelial cells (60). Through excessive activation of the NF−κB signaling pathway, MAdCAM−1 expression on liver endothelial cells increases dramatically (several−fold compared with healthy conditions), far exceeding physiological levels (61). This pathological alteration leads to two serious consequences: (1) Misguided sequestration: CD4+ T cells that would normally home to the intestine are massively retained in the liver (62). Experiments have shown that the number of CD4+ T cells retained in the liver of septic patients increases by more, while the number of such cells in the intestinal lamina propria decreases. (2) Local immune imbalance: The retained T cells release excessive pro−inflammatory cytokines (e.g., IFN−γ, IL−17), inducing neutrophil over−infiltration and oxidative stress in the liver, thereby exacerbating hepatocellular injury and creating a vicious “inflammation−damage” cycle (60). In contrast to the liver, intestinal vascular endothelial cells exhibit a sharp decrease in MAdCAM−1 expression during sepsis. The underlying mechanisms may include: (1) Hemodynamic disturbances: the intestine is one of the organs most susceptible to ischemia in early sepsis, and local hypoxia inhibits MAdCAM−1 synthesis (62); (2) Interference by inflammatory mediators: high concentrations of TNF−α can downregulate MAdCAM−1 gene expression in intestinal endothelial cells (63, 64); (3) Endothelial barrier disruption: bacterial toxins directly damage endothelial cells, causing MAdCAM−1 shedding into the bloodstream. These conditions deprive immune cells of their navigation targets. Animal experiments have confirmed that the number of T cells homing to the intestine is reduced in septic mice, directly leading to a decrease in IgA−secreting cells in the intestinal mucosa. Consequently, pathogens penetrate the intestinal barrier and enter the circulation, Gram−negative bacilli overgrow and release more endotoxin, and the intestine becomes a “factory of inflammatory factors”, accelerating systemic organ damage (65, 66).

The pathological changes in the liver and intestine mutually exacerbate each other through the “immune−metabolic axis”. The massive sequestration of T cells in the liver not only weakens intestinal defense but also contributes to systemic immunosuppression; bacteria and endotoxin that escape from the gut reach the liver via the portal vein, forcing hepatic macrophages to work excessively and release more inflammatory mediators; liver injury impairs detoxification function, and intestinal ischemia leads to lactate accumulation, together driving higher mortality (67). The “mis−navigation” of the α4β7/MAdCAM−1 pathway reveals the spatial specificity of immune dysregulation in sepsis (68).

### 3.3 Dysfunction of gut-homing regulatory T cells and immune imbalance

Sepsis, a systemic inflammatory response syndrome triggered by infection, involves not only direct pathogen-mediated damage but also a profound dysregulation of the immune system. Recently, dysfunction of gut-homing regulatory T cells (Tregs) has been identified as a core component of the immune disorder in sepsis (69). These Tregs, endowed with specific gut-homing capacity, act as an “immune brake” during the pathological progression of sepsis by modulating local intestinal immune responses and maintaining gut barrier homeostasis (70, 71). However, the sharp reduction in Treg numbers, their functional exhaustion, and the consequent imbalance with Th17 cells in sepsis directly lead to intestinal barrier breakdown and systemic inflammatory (72).

#### 3.3.1 Treg cells: numerical reduction and functional loss

Recent applications of single-cell sequencing technology have revealed the dynamic changes in Treg subpopulations during the early stage of sepsis. Within 24 hours of sepsis onset, the number of gut-homing Foxp3+CCR9+ Tregs drops sharply compared to normal conditions (73). This depletion is highly selective; compared to other Treg subsets (e.g., skin-homing CCR4+ Tregs), the gut-homing subset exhibits a significantly higher apoptosis rate (74). Further mechanistic studies indicate that pathogen-associated molecular patterns (PAMPs) induce mitochondrial pathway apoptosis in Tregs by activating the Fas/FasL signaling pathway. Experiments have shown that knockout of the Fas gene in Tregs improves their survival rate in sepsis (75, 76).

Even in Tregs that do not undergo apoptosis, their immunosuppressive function is severely impaired. Co-culture experiments have confirmed that the suppression index (the capacity to inhibit CD4+ effector T cell proliferation) of Tregs from septic patients is downregulated compared to that of healthy individuals (68). This functional defect stems from multi-level molecular disruptions: (1) Epigenetic dysregulation: increased methylation levels in the promoter region of the Foxp3 gene, leading to reduced transcriptional activity (77); (2) Metabolic reprogramming: decreased mitochondrial oxidative phosphorylation (OCR) capacity and an increased proportion of glycolysis (ECAR), which impairs the long-term survival of Tregs; (3) Signaling pathway abnormalities: reduced expression of the IL-2 receptor alpha chain, preventing Tregs from effectively receiving survival signals, along with decreased STAT5 phosphorylation levels (78). This “dual numerical and functional exhaustion” deprives Tregs of their ability to restrain effector T cells, directly resulting in uncontrolled immune responses.

#### 3.3.2 Pathological expansion of Th17 cells and disruption of the intestinal barrier

In stark contrast to Treg attenuation, Th17 cells in the gut lamina propria exhibit explosive growth in sepsis (72). Studies have shown that sepsis induces chronic, non-specifically enhanced IL-17 production by CD4+ T cells (79). Using a mouse model of LPS-induced endotoxemia, flow cytometry quantification of intestinal Th17 cells revealed a significant increase in the number of gut Th17 cells under septic conditions (80). This activation is highly specific; the magnitude of Th17 expansion in the gut is significantly higher compared to other inflammatory sites (e.g., the lungs) (81, 82).

Gut microbiota dysbiosis in sepsis is a key trigger for aberrant Th17 activation. 16S rRNA sequencing has revealed (83) that the abundance of gut Proteobacteria (e.g., Escherichia coli) is increased in septic patients. Flagellin secreted by these bacteria continuously activates dendritic cells via the TLR5/MyD88 pathway, promoting the secretion of IL-6 and IL-23, which in turn drives Th17 differentiation (84). Concurrently, the local hypoxic environment in the gut (pO2 < 20 mmHg) upregulates RORγt expression via HIF-1α, further amplifying IL-17A transcription (85).

**Figure 1:**
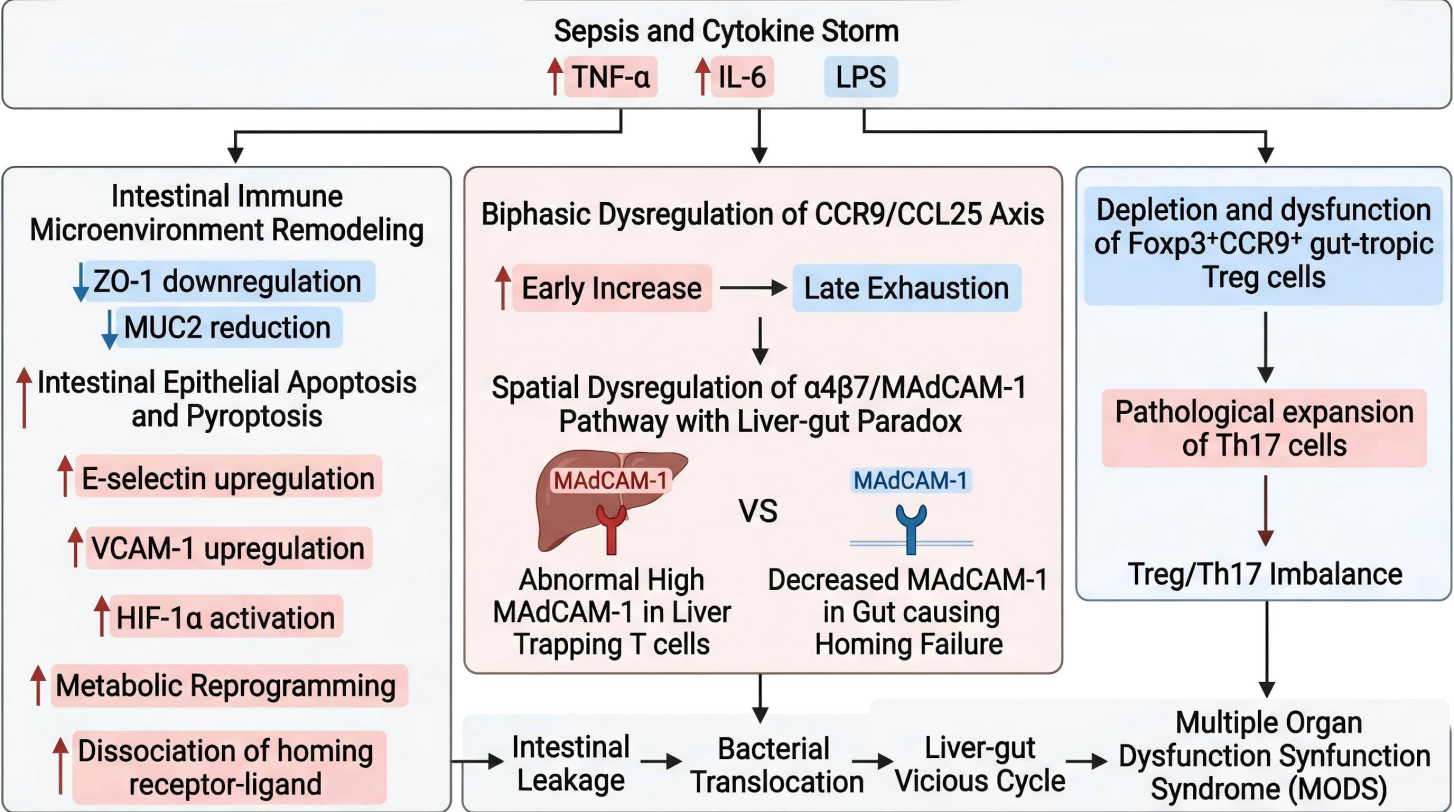
Schematic diagram of mechanisms underlying sepsis-induced intestinal lymphocyte homing disorder.

The key events involved in aberrant intestinal lymphocyte homing during sepsis are summarized in **Figure 1**.

## 4 Targeted interventional strategies for homing mechanisms

### 4.1 Chemokine receptor antagonists

In recent years, therapeutic strategies targeting chemokine receptors have gained attention in sepsis, with intestinal homing receptor CCR9 antagonists showing unique potential. As a key mediator of lymphocyte migration to the gut, CCR9 binds to CCL25 secreted by intestinal endothelial cells under physiological conditions, guiding the homing of Treg, Th17, and other cells (86). In sepsis, however, this pathway is overactivated, leading to excessive local immune infiltration in the gut and systemic lymphocyte redistribution. Recent findings suggest that precise modulation of CCR9 signaling may offer a new approach to breaking the vicious cycle of sepsis (87). In septic mouse models, CCR9 antagonists exert dual regulatory effects: (1) Suppression of intestinal inflammation—by blocking the CCR9/CCL25 interaction, they reduce intestinal CD4+ T cell infiltration and lower levels of the Th17-related cytokine IL-17A (88); (2) Improvement of circulating immunity—peripheral blood lymphocyte counts increase, and antimicrobial T cell subsets recover to near-normal levels (85, 89). Selective CCR9 blockade may reduce excessive systemic immunosuppression (90). The development of CCR9 antagonists represents a shift from broad-spectrum immunosuppression to targeted modulation in sepsis therapy (67, 91).

### 4.2 Integrin-directed regulation strategies

The core challenge in sepsis treatment is balancing excessive inflammation with immune suppression, driven by disordered integrin-mediated lymphocyte homing (92). Recent strategies focus on bidirectional modulation of α4β7 integrin—inhibiting aberrant homing or enhancing physiological migration—to restore immune cell distribution (93). Conventional integrin inhibitors (e.g., anti-α4β7 antibodies) reduce inflammatory infiltration but risk systemic immune paralysis (94). A reported nanocarrier-delivered siRNA system overcomes this: PLGA nanoparticles modified with a MAdCAM-1-mimetic peptide bind intestinal endothelium (95–97); local release of α4β7 siRNA reduces α4β7 expression in mesenteric lymph node CD4+ T cells without affecting splenic T cells (98). In septic mice, this therapy restored splenic CD4+ T cell counts, reduced intestinal damage, and avoided increased infection risk (99). Conversely, the small-molecule integrin activator AJM300 allosterically enhances α4β7–MAdCAM-1 binding, improving homing efficiency (100). This “homing enhancement” promotes Treg migration to the gut and accelerates pathogen-specific T cell arrival at infection sites (101), offering a novel approach for immune reconstitution in sepsis.

### 4.3 Microbiota-immune crosstalk intervention

The vicious cycle of gut dysbiosis and impaired immune cell homing in sepsis is a current research focus (102). Targeted modulation of the microbiota-immune network can reshape lymphocyte homing, offering an ecologically safe and immune-specific therapy for sepsis. Microbiota-derived metabolites regulate Treg differentiation and gut homeostasis (103, 104). Short-chain fatty acids enhance Treg stability by inhibiting HDAC and promoting Foxp3 acetylation. Lactobacillus reuteri activates the AhR pathway via tryptophan metabolites to modulate intestinal immunity (105, 106). In sepsis models, microbiota modulation restores the Th17/Treg balance and reduces mucosal injury. These findings suggest microbiota-directed immune modulation as a potential strategy for sepsis-induced gut barrier dysfunction, though the molecular mechanisms and clinical applications require further study (107–112).

**Figure 2:**
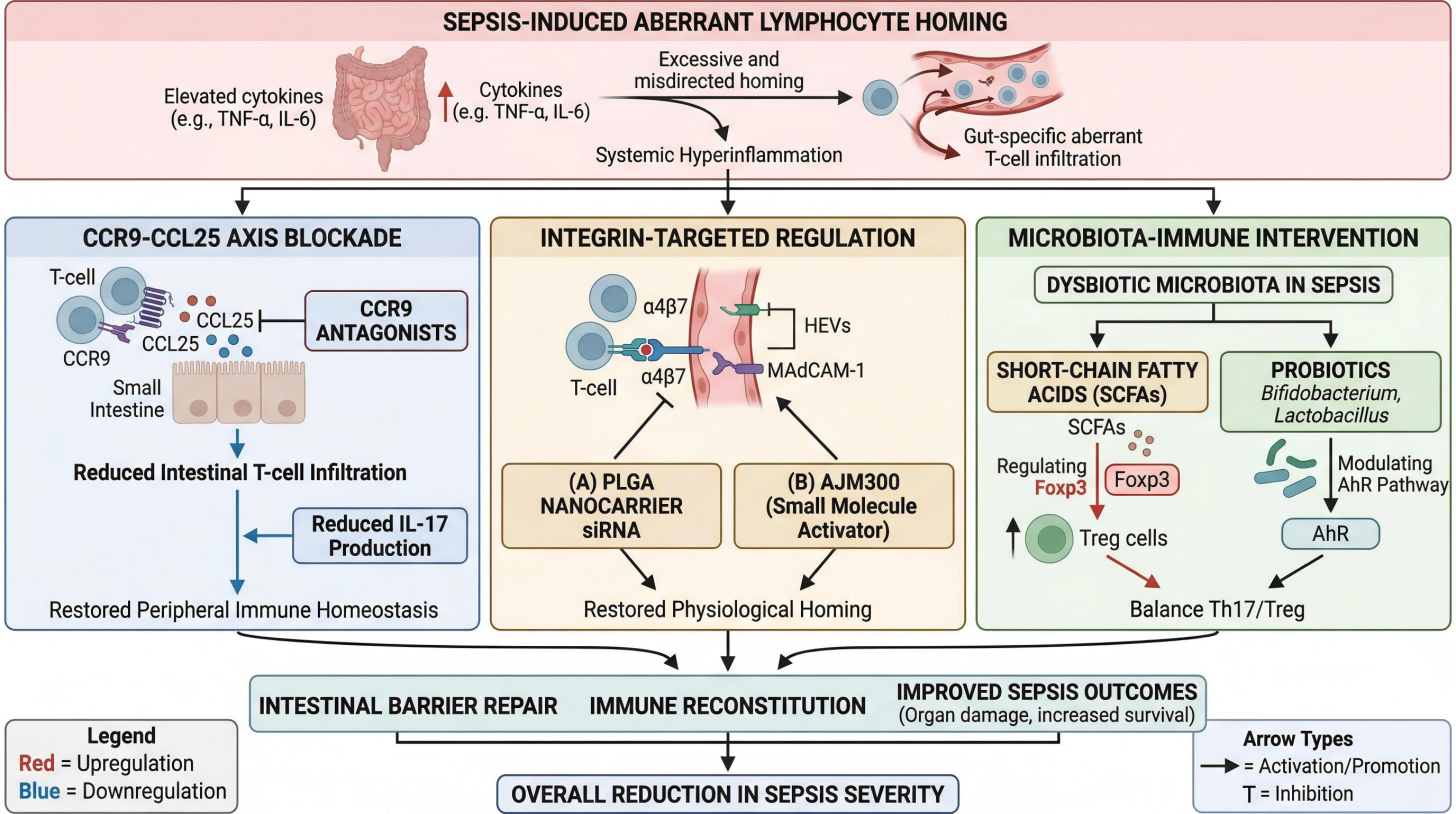
Therapeutic strategies targeting sepsis-induced aberrant intestinal lymphocyte homing.

**Figure 2** the three key therapeutic strategies targeting this pathological cascade.

## 5 Summary and outlook

In sepsis, dysregulated lymphocyte homing caused by inflammatory storm, immune exhaustion, and microcirculatory dysfunction leads to a marked reduction and functional impairment of intestinal immune cells, particularly T cells and sIgA-producing B cells. This severely compromises gut mucosal immune defense, disrupts immune tolerance and barrier repair, and—together with physical barrier injury—greatly promotes translocation of gut bacteria and endotoxins into the systemic circulation. Such translocation creates a sustained “secondary hit” that perpetuates systemic inflammation and multi-organ dysfunction, forming a vicious cycle that is a key factor in poor prognosis and refractory sepsis. Currently, several critical scientific questions remain unresolved: the dynamic changes of homing axes at different sepsis stages, the mechanisms of tissue-specific recognition, whether non-intestinal organs ectopically express MAdCAM-1 under inflammatory conditions, the compensatory mechanisms of the homing molecular network, the impact of sepsis heterogeneity, the relationship between immunometabolic reprogramming and homing, and the lack of stage-specific intervention strategies. Regarding targeted therapy, major challenges include balancing early inhibition versus late promotion of homing, the risk of systemic administration interfering with normal immune surveillance and increasing secondary infections, the need for coordinated antimicrobial therapy, and adherence to precision medicine principles (“right time, right state, right intervention”). To address these, it is necessary to establish a homing marker-based classification system, design stage-specific intervention strategies (e.g., using antagonists such as anti-α4β7 antibody or CCR9 antagonists in the early phase, and agonists or cytokines in the late phase), learn from failed clinical trials of CCR9 antagonists, conduct biomarker-driven adaptive trials, and explore improving microcirculation to influence capture bond-mediated homing. Therefore, understanding and modulating disordered lymphocyte homing may represent a key research direction for improving intestinal immune barrier function, reducing secondary infections and organ failure risk, and enhancing prognosis in sepsis. Specific strategies include modulating homing receptor/ligand expression, improving microcirculation, and implementing immunomodulatory therapies. The gut is not only a victim of sepsis but also an engine driving its progression, with disordered lymphocyte homing serving as a critical immunopathological link in this process.
